# Malaria case management in Papua New Guinea following the introduction of a revised treatment protocol

**DOI:** 10.1186/1475-2875-12-433

**Published:** 2013-11-27

**Authors:** Justin Pulford, Serah F Kurumop, Yangta Ura, Peter M Siba, Ivo Mueller, Manuel W Hetzel

**Affiliations:** 1 Papua New Guinea Institute of Medical Research (PNGIMR), Goroka, EHP 441, Papua New Guinea; 2 The University of Queensland, School of Population Health, Herston, Qld 4006, Australia; 3 Barcelona Centre for International Health Research (CRESIB Hospital Clínic-Universitat de Barcelona), Barcelona, Spain; 4 Walter and Eliza Hall Institute of Medical Research, Melbourne, Australia; 5 Swiss Tropical and Public Health Institute, PO Box, 4002, Basel, Switzerland; 6 University of Basel, Petersplatz 1, 4003 Basel, Switzerland

## Abstract

**Background:**

This paper reports on the availability of diagnostic tools and recommended anti-malarials in the 12-month period immediately following the implementation of a new national malaria treatment protocol (NMTP) in Papua New Guinea (PNG). Health worker adherence to the new NMTP is also examined and comparisons made with previously reported pre-implementation findings.

**Methods:**

A countrywide cross-sectional survey in randomly selected primary health care facilities (n = 88). Data were collected via passive observation of the clinical case management of fever or suspected malaria patients and via an interviewer administered questionnaire completed with the officer in charge of each participating health care facility.

**Results:**

Malaria rapid diagnostic tests (RDTs) and the new first-line anti-malarial medication, artemether-lumefantrine (AL), were available in 53.4% and 51.1% of surveyed heath facilities, respectively. However, they were more widely available in the larger health centres as compared to the smaller aid-posts (90.2% vs. 21.3% and 87.8% vs. 19.2%, respectively). Overall, 68.3% of observed fever cases (n = 445) were tested for malaria by RDT and 39% prescribed an anti-malarial, inclusive of 98.2% of RDT positive patients and 19.8% of RDT negative cases. The availability and use of malaria RDTs was greater in the current survey as compared to pre-implementation of the new NMTP (8.9% vs. 53.4% & 16.2% vs. 68.3%, respectively) as was the availability of AL (0% vs. 51.1%). The percentage of fever patients prescribed anti-malarials decreased substantially post implementation of the new NMTP (96.4% vs. 39.0%).

**Conclusions:**

PNG has achieved high coverage of malaria RDTs and AL at the health centre level, but these resources have yet to reach the majority of aid-posts. Malaria case management practice has substantially changed in the 12-month period immediately following the new NMTP, although full protocol adherence was rarely observed.

## Background

Papua New Guinea (PNG), a malaria-endemic country of approximately seven million people in the South Pacific, introduced a new national malaria treatment protocol (NMTP) in late 2011. Consistent with the recommendations of the World Health Organization [1], the new NMTP stipulates that all fever or suspected malaria cases be tested for malaria infection by microscopy or rapid diagnostic test (RDT), introduces artemether-lumefantrine (AL) as the new first-line treatment for uncomplicated *Plasmodium falciparum* malaria, AL plus primaquine (PQ) as the new first-line treatment for uncomplicated *Plasmodium vivax* malaria and artesunate injection followed by AL for first-line treatment of severe *P. falciparum* malaria, with the addition of PQ for the treatment of severe *P. vivax* malaria [2]. Dihydroartemisinin-piperaquine was also introduced as the new second-line treatment for uncomplicated *P. falciparum* and *P. vivax* malaria, with the addition of PQ for the latter.

A national cross-sectional survey conducted prior to the implementation of the new NMTP identified malaria RDTs or functional microscopy in only 15% of health facilities [3]. The lack of diagnostic capacity was evident in malaria case management practice, with fewer than 20% of 468 observed fever cases tested for malaria infection by microscopy or RDT [4], although this rose to 40.9% when the analysis was restricted to fever cases managed in health facilities with access to malaria RDTs or functional microscopy. Anti-malarial prescription was near universal with 96.4% of the 468 observed fever cases prescribed an anti-malarial, including 82% of the 50 patients who tested negative for malaria infection by microscopy or RDT [4]. Overall, 79.8% of these prescriptions conformed to the treatment protocol current at that time.

These baseline findings indicate a substantial change in malaria diagnostic capacity and malaria case management practice will be required if the new NMTP is to be successfully implemented in PNG. In particular, access to malaria RDTs or functional microscopy will need to improve and health workers will need to utilize malaria RDTs or microscopy at greater rates when they are available. As recent evidence indicates that many fever cases presenting to health facilities in PNG are non-malarial in origin [5], then adherence to the new protocol will likely see rates of anti-malarial prescription reduce dramatically e.g. [6]. The international experience suggests these changes in health worker practice will take some time to achieve. For example, three years after the implementation of a protocol stipulating routine microscopy or RDT testing of all adult fever cases and the prescription of AL to test positive cases in Kenya, testing rates in health facilities with RDT or microscopy available did not exceed 54% and nearly a third of test negative cases were prescribed AL [7]. Similar accounts of poor health worker adherence and other institutional barriers to the implementation of an RDT/artemisinin combination therapy-based malaria treatment policy have been documented elsewhere [8,9], including neighbouring Timor-Leste [10].

In order to assess implementation progress in PNG, this paper reports on the availability of microscopy, malaria RDT, AL and other recommended anti-malarials in the 12-month period immediately following introduction of the new NMTP, as well as health worker adherence to the recommended diagnostic and prescription practices. Comparisons in resource availability and malaria case management practice are made with previously reported pre-implementation findings.

## Methods

### Study setting

PNG is thought to have one of the highest burdens of malaria outside of Africa. A countrywide household survey conducted in 2008/2009 reported general population malaria prevalence of 18.2% [11] with the National Department of Health reporting 1,431,395 suspected malaria cases and 604 malaria attributable deaths in 2009 [12]. A 2008 study of 1807 febrile patients presenting to five sentinel health facilities across PNG reported a malaria slide positivity rate of 45.4% [5], although marked variation ranging from 2.2% to 74.9% across sites was observed. However, more recent evidence indicates a decreasing malaria burden. Following two rounds of free countrywide distributions of long lasting insecticidal mosquito nets, general population prevalence of malaria had reduced to 6.7% in 2011 [11] and a 50% reduction in clinical incidence between was reported in East Sepik province [13].

### Study procedures

The study procedures for the health facility survey reported in this paper are consistent with a 2010 survey previously described [3,4]. A detailed description of the evaluation of the PNG National Malaria Control Programme, of which the health facility surveys are one of several components, is also available in the published literature [14]. The following description of the survey methodology is a summarized version of these previously published accounts.

At the time of drafting the health facility survey methodology, PNG consisted of 20 provinces divided into four geographical regions (Southern, Highlands, Momase, and Islands). The study sample consisted of two health centres or health subcentres (collectively referred to as health centres in this paper) and up to four aid posts selected from each of these 20 provinces, using a simple random sampling procedure. The sampling frame was a list of all operational public-sector health centres nationwide as provided by the National Department of Health (N = 689; the small number of private sector health facilities were excluded from survey). Aid posts were randomly selected on site at participating (i e, randomly selected and consenting) health centres. The sampling frame for aid posts was all operational aid posts under the supervision of the health centre at the time of survey.

The study was carried out from June to November 2012 and was conducted by three trained field teams working simultaneously at different sites. Members of each survey team spent between three to five days at each participating health centre and up to one day at each participating aid post. Four survey instruments were completed at each health facility, although this paper only reports data obtained from two of these: a structured checklist completed with the officer in charge of the participating health facilities, designed to assess the availability of supplies relevant to malaria case management; and, a structured checklist designed to record observed features of the clinical case management of patients presenting with fever or a recent history of fever. The remaining instruments included interviewer administered questionnaires completed with health workers and with febrile patients following service discharge. Information obtained from these questionnaires primarily centered on barriers to (health worker interviews), and the quality of (patient interviews), service provision and will be reported separately. Oral informed consent was sought from the officer in charge at all participating health facilities and from all participating clinicians and patients prior to clinical observation. The study was approved and granted ethical clearance by the Medical Research Advisory Committee of PNG (MRAC No. 10.12; 26 Feb 2010).

### Data analysis

All data were double entered into DMSys version 5.1 (Sigma Soft International). Stata/SE version 12 was used for descriptive data analysis, for calculating 95% confidence intervals (CIs) and for comparing differences in malaria resource availability and malaria case management between health facility types and/or pre- and post-NMTP implementation (Pearson chi square). A complete description of pre-NMTP implementation data can be found elsewhere [3,4]. The calculation of all CIs reported on the non-participant observation sample was adjusted for possible clustering at the health facility level by using the Stata ‘svy’ command in which health facilities were defined as the primary sampling unit.

## Results

### Resource availability

A total of 88 health facilities were included in the survey. Of these, 41 were health centres and 47 were aid posts (Table 1).

**Table 1:** Surveyed health facilities by type and region

**Health facility type**	**Region**				**Total**
	**Southern**	**Highlands**	**Momase**	**Islands**	
Health centre	15	11	8	7	41
Aid post	16	11	10	10	47
Total	31	22	18	17	88

The availability of diagnostic tools and medications necessary for implementation of the revised NMTP is presented in Table 2. As shown, RDTs were more widely available than microscopy and the small number of health facilities that had functional microscopy available, also had RDTs in stock. An additional seven health facilities had a microscope in stock, but did not meet the study definition of functional microscopy: the presence of a working microscope, all essential supplies (Giemsa stain, slides and, in the case of electric microscopes, power) and a trained microscopist in employment.

**Table 2:** Percentage of health facilities with the required resources for implementation of the new national malaria treatment protocol

**Resource**	**Health centre**		**Aid post**		**Overall**	
	**%**	**(95% CI)**	**%**	**(95% CI)**	**%**	**(95% CI)**
*Diagnostic Test*						
RDT	90.2	(76.9, 97.3)	21.3	(10.7, 35.7)	53.4	(42.5, 64.1)
Microscopy^a,^	7.3	(1.5, 19.9)	2.1	(<0.1, 11.3)	4.6	(1.3, 11.2)
RDT or microscopy	90.2	(76.9, 97.3)	21.3	(10.7, 35.7)	53.4	(42.5, 64.1)
*Recommended medication – first- and second-line treatment*^*b*^						
AL^c^, all categories	87.8	(73.8, 95.9)	19.2	(9.1, 33.3)	51.1	(40.2, 61.9)
AL, 5–15 kg	95.1	(83.5, 99.4)	21.3	(10.7, 35.7)	55.7	(44.7, 56.3)
AL, 15-25 kg	92.7	(80.1, 98.5)	23.4	(12.3, 38.0)	55.7	(44.7, 56.3)
AL, 25–35 kg	92.7	(80.1, 98.5)	19.2	(9.1, 33.3)	53.4	(42.5, 64.1)
AL, 35+ kg	92.7	(80.1, 98.5)	23.4	(12.3, 38.0)	55.7	(44.7, 56.3)
AL + PQ^d^	68.3	(51.9, 81.9)	14.9	(6.2, 28.3)	39.8	(29.5, 50.1)
DP^e^	4.9	(<0.1, 16.5)	0	-	2.3	(<0.1, 8.0)
AI + AL^f^	70.7	(54.5, 83.9)	8.5	(2.4, 20.4)	37.5	(27.4, 48.5)
AI + AL + PQ^g^	61.0	(44.5, 75.8)	8.5	(2.4, 20.4)	33.0	(23.3, 43.8)
QI + QT + DX^h^	65.9	(49.4, 79.9)	29.8	(17.3, 44.9)	46.6	(35.9, 57.5)
*Selected ‘other’ anti-malarial medication*						
SP	97.6	(87.1, 99.9)	87.2	(74.3, 95.2)	92.1	(84.3, 96.7)
CQ	92.7	(80.1, 98.5)	85.1	(71.7, 93.8)	88.6	(80.1, 94.4)
AQ	92.7	(80.1, 98.5)	87.2	(74.3, 95.2)	89.8	(81.5, 95.2)
Artemether tablets	68.3	(51.9, 81.9)	42.6	(28.3, 57.8)	54.6	(43.6, 65.2)

^a^Microscopy was defined as the presence of a functional microscope, all essential supplies (Giemsa stain, slides and, in the case of electric microscopes, power) and a trained microscopist in employment.

^b^The quantity of each medication was not accounted for in this analysis; rather, the data represent the percentage of health facilities that had at least one vial or container (inclusive of a single, opened container) of the respective anti-malarial in stock.

^c^First-line treatment for uncomplicated *P. falciparum* infection.

^d^First-lline treatment for uncomplicated *P. vivax* infection.

^e^Second-line treatment for uncomplicated malaria infection.

^f^First-line treatment for severe *P. falciparum* infection.

^g^First-line treatment for severe *P. vivax* infection.

^h^Second-line treatment for severe malaria infection.

AL = artemether-lumefantrine, PQ = primaquine, DP = dihydroartemisinin-piperaquine, AI = artemether or artesunate injection, QI = quinine injection, QT = quinine tablets, DX = doxycycline, SP = sulphadoxine-pyrimethamine, CQ = chloroquine, AQ = amodiaquine.

The brand of RDT kit was recorded in 35 health facilities, the majority of which had the ICT malaria combo test (n = 33). CareStart™ Malaria brand RDTs was present in one health facility and both ICT and CareStart™ in a further health facility. Resource availability was substantially greater at the health centre level as compared to the aid post level in nearly all cases. The difference in availability of RDTs and AL, the two resources most commonly required in the implementation of the new protocol, between the health facility types, reached a level of statistical significance (χ^2^ = 41.857, p < 0.001; χ^2^ = 41.309, p < 0.001, respectively). Overall, a total of 27,777 unexpired RDT kits were observed across the health facility sample compared to a total of 53,677 unexpired AL doses. Of the 47 health facilities with either AL or RDT in stock, 80.9% (38/47) had more AL than RDT.

The availability of RDTs increased from 8.9 to 53.4% (χ^2^ = 37.7587, p < 0.001) across all health facilities between 2010 (previously reported pre-implementation survey) and 2012 and from 17.5 to 90.2% (χ^2^ = 43.1785, p < 0.001) and 0 to 21.3% (χ^2^ = 9.3897, p = 0.002) at the health centre and aid post levels, respectively. AL was not available at any health facility in the 2010 survey.

### Malaria case management

A total of 556 clinical observations were completed with patients presenting with fever or a recent history of fever during the survey period. Patients who had been treated for fever or malaria infection within 14 days prior to interview were subsequently removed from analysis to ensure the findings better represented initial malaria case management practice. This restriction resulted in a final sample of 445 clinical observations obtained from 43 health facilities. Only 1.4% (6/445) of these patients were treated at an aid post, an outcome which reflects the brief amount of time the research teams spent at aid-posts relative to health centres and the fact that a single aid post receives fewer patients than a single health centre (although aid posts are more numerous than health centres so still account for a substantial number of outpatient cases per year). Table 3 presents sex and age characteristics of the observed fever patients by region.

**Table 3:** Sex and age of the clinical observation sample by region (n = 445)

**Characteristic**		**Region**				**Overall**
		**Southern**	**Highlands**	**Momase**	**Islands**	
Female n (%)		70 (51.1)	54 (47.4)	67 (52.8)	48 (71.6)	239 (53.7)
Age n (%)	0–4 yrs	57 (41.6)	51 (44.7)	62 (48.8)	26 (38.8)	196 (44.0)
	5–15 yrs	34 (24.8)	15 (13.2)	29 (22.8)	20 (29.9)	98 (22.0)
	16+ yrs	46 (33.6)	48 (42.1)	36 (28.4)	21 (31.3)	151 (33.9)

### Use of RDT or microscopy

Overall, 68.3% (304/445; 95% CI 52.0, 81.1) of the observed fever patients were tested for malaria infection by RDT. When restricted to health facilities that had RDT in stock, this increased to 73.3% (280/382; 95% CI 55.7, 85.7). A blood slide was not taken from any of the observed fever patients. No statistically significant difference was observed in the percentage of patients tested for malaria infection by age (<five years *vs* five + years) or sex (female *vs* male) in those health facilities with RDT in stock (74.5 *vs* 72.4%; χ^2^ = 0.2975, p = 0.862 and 72.9 *vs* 73.8%; χ^2^ = 1.6505, p = 0.438, respectively).

The use of RDTs or microscopy in malaria case management increased by a statistically significant level across all health facilities between 2010 and 2012 (16.2 *vs* 68.3% χ^2^ = 253.2963, p < 0.001), including when the analysis was restricted to only those health facilities that had RDT or microscopy available (41.5 *vs* 73.3%, χ^2^ = 43.6338, p < 0.001).

### Anti-malarial prescription

Prescription information was available for 98.4% (438/445) of the observed fever patient sample. Table 4 presents the number and percentage of these patients prescribed an anti-malarial (any anti-malarial and AL) by diagnostic category (no RDT, positive RDT result, negative RDT result) and overall. As can be seen, 39% (171/438) of the observed fever patients were provided any anti-malarial medication and 12.8% AL. Of the patients tested for malaria infection by RDT, 98.2% of test positive cases were prescribed an anti-malarial as were 19.4% of test negative cases. All except one of the test positive cases prescribed an anti-malarial were given AL compared to fewer than 1% of test negative cases.

**Table 4:** Number and percentage of observed fever patients prescribed an anti-malarial by diagnostic category and overall

**Diagnostic category**	**No (%)**	**Any antimalarial**		**Artemetherlumefantrine**	
		**%**	**95% CI**	**%**	**95% CI**
No RDT	136 (31)	51.5	(28.6, 73.8)	1.5	(<0.1, 11.4)
Positive RDT	54 (12)	98.2	(84.1, 99.8)	98.2	(84.1, 99.8)
Negative RDT	248 (57)	19.4	(9.6, 35.1)	<1	(<0.1, 2.8)
**Overall**	**438 (100)**	**39.0**	**(27.8, 51.7)**	**12.8**	**(7.4, 21.3)**

Of the 98.5% (53/54) RDT-positive patients who were prescribed AL, 41.5% (22/53) tested positive for “*P. falciparum* mono-infection or mixed infection”, 35.8% (19/53) for “*P. falciparum*”, 17.0% (9/53) for “non-falciparum” (*P. vivax*, *P. ovale*, *P. malariae* or a mixed infection of these) and in 5.7% (3/53) cases the species result was not recorded. According to the NMTP, patients who test positive for a mixed infection or a non-*P. falciparum* infection should also be prescribed primaquine. This occurred in 51.6% (16/31) of these cases. Chloroquine was prescribed in addition to primaquine in one mixed infection case and primaquine was prescribed to one *P. falciparum*-infected patient. No other anti-malarials were prescribed to these patients, although some form of other medication was provided in 51.9% (28/54) of RDT-positive cases. These other medications included analgesics (n = 23), antibiotics (n = 8), antihelminthics (n = 7) and anti-anaemia (n = 2).

Of the RDT negative or presumptively diagnosed patients prescribed anti-malarials, 75% (87/116) were prescribed the obsolete first-line treatment for uncomplicated malaria (either amodiaquine and sulphadoxine/pyrimethamine (SP) or chloroquine and SP). The remaining patients were prescribed amodiaquine (n = 12), artemether tablets and SP (n = 5), quinine tablets and SP (n = 2), artemether tablets (n = 2), SP (n = 2), AL (n = 2), artesunate tablets and SP (n = 1), chloroquine, SP and primaquine (n = 1), artemether tablets, SP and primaquine (n = 1) or chloroquine, SP and quinine (n = 1). Overall, 84.5% (98/116) of these patients were observed in a health facility which had all doses of AL in stock. Some other form of medication was provided in 79.3% (92/116) of cases. These other medications included antibiotics (n = 71), analgesics (n = 65), antihelminthics (n = 14), antiprotozoals (n = 2), anti-anaemia (n = 2) and gastro-intestinal medication (n = 1). Patients prescribed an anti-malarial either presumptively or with a negative RDT result were more likely to be prescribed another form of medication compared to RDT-positive patients prescribed an anti-malarial (79.3 *vs* 51.9%; χ^2^ = 17.2258, p < 0.001).

In terms of compliance with the NMTP prescription regimen, 19.3% (33/171) of the patients prescribed an anti-malarial were prescribed the correct first-line medication for uncomplicated malaria infection (defined as a prescription of AL to *P. falciparum* infection or AL and primaquine to a potentially mixed or non-*P. falciparum* infection). A further five patients (2.9%) were prescribed AL presumptively, which may be considered a correct prescription in the absence of an RDT or microscopy result.

Anti-malarial prescription to any patient decreased by a statistically significant level across all health facilities between 2010 and 2012 (96.4 *vs* 39.0%; χ^2^ = 336.8467, p < 0.001). The prescription of anti-malarial medication consistent with extant guidelines also decreased by a statistically significant margin across these two time periods (79.8 *vs* 22.2%; χ^2^ = 161.5372 p < 0.001), primarily as a result of the continued prescription of the former (obsolete) first-line anti-malarials to RDT negative and presumptively diagnosed patients.

### Treatment counselling

The percentage of fever patients observed to have been provided with each of six different ‘types’ of clinical instruction by their respective clinician(s) is presented in Table 5. The sample was restricted to patients who had been prescribed anti-malarial medication. As shown, the dosage instructions and the purpose of the medication supplied were explained in the majority of cases (86 and 64.9%, respectively). The remaining instructions were all provided in fewer than 25% of cases, with an explanation of possible adverse effects discussed in 2.3% of cases.

**Table 5:** Observed provision of instructions to patients prescribed anti-malarial medication (n = 171)

**Instruction**	**% provided**	**(95% CI)**
Purpose of medication	64.9	(57.3, 72.0)
Dosage/regimen	86.0	(74.8, 92.7)
Dietary	17.5	(10, 29)
Possible adverse effects	2.3	(0.6, 5.9)
Health facility re-engagement^a^	18.1	(12.7, 24.7)
Prevention advice	19.3	(13.7, 26.0)

^a^In which patients are advised to return to the health facility if current symptoms persist or deteriorate.

When compared to the previously reported 2010 findings, statistically significant increases in the percentage of patients receiving the following forms of clinical instruction were evident: dosing regimen (75.7 *vs* 86%; χ^2^ = 8.2609, p = 0.004); dietary advice (6.2 *vs* 17.5%; χ^2^ = 19.4929, p < 0.001); and malaria prevention advice (10.3 *vs* 19.3%; χ^2^ = 9.2663, p = 0.002). A statistically significant decrease in the percentage of patients receiving instruction on when to return to the health facility was observed (27.7 *vs* 18.1%; χ^2^ = 4.9317, p = 0.026). Changes in the percentage of patients receiving the remaining instructions did not reach a level of statistical significance: purpose of medication (63.4 *vs* 64.9%; χ^2^ = 0.7739, p = 0.379) and explanation of possible side effects/adverse events (1.1 *vs* 2.3%; χ^2^ = 1.1796, p = 0.277).

## Discussion

The reported findings indicate that RDTs are widely available at the health centre level of service provision in PNG as are all four weight packs of AL (90.2 and 87.8% of surveyed health centres, respectively). These diagnostic and treatment resources were available in fewer than 20% of health centres prior to the introduction of the new NMTP [3], indicating widespread coverage has been achieved in a relatively brief period of time. However, other anti-malarial medications required in the new NMTP, such as primaquine, artesunate and particularly dihydroartemisinin-piperaquine, were less available; the available quantity of RDTs relative to AL dosages appeared insufficient and the availability of all recommended diagnostic tools and anti-malarial medications remained low at the aid post level. Thus, the vast majority of health centres were adequately equipped to treat uncomplicated *P. falciparum* infection according to the new NMTP, but were less well equipped to treat uncomplicated *P. vivax* infections, severe malaria infections or to provide second-line treatments. Fewer than 20% of aid posts were equipped to treat any form of malaria infection in a manner consistent with the new NMTP. As aid posts comprise more than 70% of all heath care facilities [15], the lack of RDTs and AL at this level of health care is of concern.

Nearly three-quarters of observed febrile or suspected malaria patients were tested for malaria infection by RDT in those facilities that had the capacity to do so, a statistically significant increase from 40.9% in such facilities pre-implementation of the new NMTP [4]. Over 98% of patients with RDT-confirmed malaria were prescribed an anti-malarial, and in all cases this included AL, the recommended blood-stage medication for uncomplicated malaria infection. Fewer than 20% of malaria RDT-negative cases were prescribed an anti-malarial, although fewer than 1% of these anti-malarial prescriptions were AL as were fewer than 1% of anti-malarial prescriptions to presumptively diagnosed patients. Rather, the vast majority of anti-malarial prescriptions (75%) to RDT-negative and presumptively diagnosed ‘malaria’ patients were for the first-line medications of the obsolete treatment protocol (amodiaquine and SP or chloroquine and SP). Encouragingly, artemether monotherapy were only prescribed in two cases even though artemether tablets were available in over 50% of health facilities surveyed.

The prescription of obsolete or non-recommended anti-malarials to patients diagnosed with malaria, even when recommended artemisinin combination therapy are available, has been widely reported in the international literature [16]. However, one questions why a health worker would not prescribe the most effective anti-malarial medication available (eg, AL) if he/she held firm to a clinical diagnosis of malaria infection even in the face of a negative RDT/blood slide. A study from Tanzania found that local illness labels for febrile illness, as opposed to biomedical classifications, influenced anti-malarial prescription [17]. As there is some evidence that local understanding of malaria may differ to biomedical classification in PNG [18,19], then health worker practice in the study setting may have been similarly influenced. A further influence may have been the NMTP training programme itself. This programme strongly promoted a strict test-and-treat with AL strategy, an unexpected consequence of which may have been reluctance on the part of health workers to prescribe AL when RDTs were not available or used or when clinical judgement was used to prescribe anti-malarials despite a negative RDT result.

Just under 40% of all observed febrile or suspected malaria patients were prescribed an anti-malarial. This represents a statistically significant decline in anti-malarial prescription in this population, from a pre-implementation rate of 96%, and indicates the new NMTP has affected a substantial change in clinical practice in a relatively brief period of time. Nevertheless, the majority of anti-malarial prescriptions made were not consistent with the new NMTP and it remains questionable as to whether such a dramatic reduction in anti-malarial prescription is clinically justified. Thus, further investigation is needed to better understand clinical decision-making in the absence of malaria diagnostic tests and in the treatment of malaria RDT/blood slide test negative cases. The reported findings also suggest that the quality of treatment counselling is relatively poor, despite emphasis in the new NMTP and the associated health worker training programme [2,20].

Whilst far from perfect, these findings compare well with the international experience. In Zambia, for example, health workers diagnosed fewer than 30% of fever patients with a malaria RDT/blood slide following the introduction of a treatment protocol similar to that employed in PNG, even when RDTs/microscopy were available [8]. Similarly, numerous studies have reported anti-malarial prescription to between 30 and 50% of malaria RDT-negative cases [8,21,22], even several years following the introduction of RDT kits [7]. Seen in this light, health workers in PNG may be considered to have made encouraging progress towards full protocol adherence within the first 12 months of implementation. Factors that may have influenced this outcome included an independent procurement and supply mechanism established specifically for the NMCP, the recruitment of numerous malaria supervisors and laboratory technicians tasked with supporting the implementation of the new NMTP at the provincial level, booster training where required and dedicated funding to support these initiatives.

Nevertheless, substantial improvement in many areas is required before a sustained and full implementation of the new NMTP may be considered achieved. Increased coverage of RDTs and all recommended first- and second-line anti-malarial medications is needed, especially at the aid post level. The ability of the PNG National Department of Health’s procurement and supply mechanism to maintain adequate RDT and anti-malarial supplies, once the independent system is phased out, remains untested at this stage. In the context of PNG’s declining malaria burden [11], and consequently increasing proportion of malaria-negative fever cases, supply needs of both diagnostics and treatment will require constant re-assessment based on routine surveillance data. Again, experience from other countries would suggest that supply stock-out presents a serious threat to protocol implementation [23] and needs to be closely guarded against.

Further reducing the percentage of malaria RDT/blood slide negative cases prescribed anti-malarials remains important. Treating malaria based on RDT diagnosis has been proven safe in areas with moderate to high endemicity of both *P. falciparum* and *P. vivax* infections in PNG [24]. Thus, the local evidence is available to support the non-prescription of anti-malarials to test negative cases. The unnecessary prescription of anti-malarials may accelerate the development of artemisinin resistance in PNG, especially if artemether monotherapy are prescribed [25], reduces the cost-effectiveness of the test-and-treat-based malaria case management policy [26] and may compromise patient wellbeing [27]. Providing further reassurance to health workers regarding the reliability of malaria RDT diagnosis may, therefore, be required. Similarly, health workers need to be actively encouraged to prescribe recommended anti-malarials at all times and in all cases when malaria is diagnosed, even presumptively. Health workers are likely to require long-term assistance, ideally via multiple support and supervisory mechanisms, in order for such substantial and sustained changes in malaria case management practices to occur [28].

Advancing understanding of common causes of non-malaria febrile illness in PNG would usefully inform health worker practice, although few local studies have been conducted to date. A recent exception sought to identify the aetiology of febrile illnesses among patients (n = 136) attending a health centre in Western province, PNG [29]. An aetiological agent was found in only 13.2% of these patients; 11% were dengue virus type 1 and 2.2% malaria. An earlier study from Madang province, PNG, reported a similar rate of dengue infection (8%) among febrile patients attending local health centres [30]. These findings suggest dengue is likely to be a cause of febrile illness in many places across the country, although it is unlikely to be a major cause of non-malaria febrile illness and the aetiology and prevalence of other febrile illnesses remains uncertain. A recent outbreak of chikungunya in PNG [31] further indicates that disease profiles are not static and that health workers need to be adequately equipped to correctly identify and respond to a wide range of existing and emerging febrile illnesses.

The reported study was not without limitation. The health facility survey was conducted during a period of lower malaria transmission (June-November) in those provinces with seasonal variation. Thus, the number of malaria patients presenting to health facilities and the subsequent pressure on resources (eg, RDT kits, anti-malarial medication) may have been lower during the survey period as opposed to peak transmission periods. It is also possible that health workers may treat patients differently in low and high transmission seasons depending on what they perceive the most likely cause of fever to be. Participating clinicians were aware that they were being observed and may have altered their clinical practice accordingly. The expected effect of any such bias would be towards perceived ‘better’ practice. The sample excluded private-sector health facilities and, as such, may not be representative of resource availability and malaria case management in these settings. However, the vast majority of health services are provided in a public-sector context in PNG and the surveyed sample is considered generally reflective of the range, geographical spread and quality of public-sector health centres and aid-posts in the country. The major limitation in terms of sampling was the lack of clinical case management data collected from aid-posts. The clinical case management data reported in this paper, therefore, should only be considered reflective of practice at the health centre level. Finally, data analysis pertaining to ‘correct’ anti-malarial prescription was based on medication type and did not take dosage into account.

## Conclusions

The findings presented in this paper suggest that the PNG National Department of Health (NDoH) has achieved reasonably high coverage of malaria RDTs and AL at the health centre level and that health workers have made an encouraging start in implementing the new NMTP. Nevertheless, many of the medications essential for full implementation of the NMTP are not widely available, the ability of the NDoH to maintain supplies has yet to be tested and further changes in health worker practice are required. In particular, health workers continue to prescribe obsolete anti-malarials at a higher rate than the new first- and second-line medications, including 20% of malaria RDT/blood slide test negative cases. Gaining a better understanding of the causes of non-malaria fever may usefully inform health worker practice as would reassurance as to the reliability of RDTs and the effectiveness of the new first- and second-line anti-malarial medications.

## Competing interests

The authors declare that they have no competing interests.

## Authors’ contributions

JP coordinated the study, conducted the analysis and drafted the final manuscript. SFK managed the field teams, contributed to data collection and critically revised the manuscript. YU created the study database, coordinated data management and prepared the final dataset for analysis. MWH conceived of the study and with PMS and IM contributed to study design and critical revision of the manuscript. All authors read and approved the final manuscript.
